# Bone formation induced by BMP-2 in human osteosarcoma cells

**DOI:** 10.3892/ijo.2013.2030

**Published:** 2013-07-23

**Authors:** LIN WANG, PAUL PARK, FRANK LA MARCA, KHOI THAN, SHAYAN RAHMAN, CHIA-YING LIN

**Affiliations:** 1 Spine Research Laboratory, Department of Neurosurgery, University of Michigan Medical School, Ann Arbor, MI, USA; 2 Department of Biomedical Engineering, University of Michigan, Ann Arbor, MI, USA

**Keywords:** BMP-2, osteosarcoma, Smad, MAPK, osteogenic differentiation, bone formation

## Abstract

Our previous studies demonstrated that BMP-2 inhibits the tumorigenicity of cancer stem cells identified as cells with high aldehyde dehydrogenase activity (ALDH ^br^ cells) from the human osteosarcoma cell line OS99-1. We further investigated whether BMP-2 is capable of inducing bone formation in OS99-1 cells. Flow cytometry sorting was used to isolate tumorigenic ALDH ^br^ and non-tumorigenic ALDH ^lo^ cells. qRT-PCR was used to quantify the gene expression. A xenograft model was used to verify the bone formation *in vivo* . There was significantly higher mRNA expression of BMPR1B and BMPR2 in ALDH ^lo^ cells compared with that in ALDH ^br^ cells and the BMPR1B expression in ALDH ^lo^ cells was ∼8-fold higher compared to that in ALDH ^br^ cells. BMP-2 was also found to induce higher transcription of osteogenic markers Runx-2, Osterix (Osx), alkaline phosphatase (ALP) and collagen type I in ALDH ^lo^ cells compared to ALDH ^br^ cells, which were mediated by the canonical Smad signaling pathway. *In vivo* , BMP-2 was identified to induce bone formation in both ALDH ^br^ and ALDH ^lo^ cells. All animals receiving 1×10 ^4^ ALDH ^lo^ cells treated with 30 *μ* g of BMP-2 per animal showed bone formation within 1–2 weeks after injection in mice. Bone formation induced by BMP-2 in ALDH ^lo^ cells showed significantly more bone mineral content compared to that in ALDH ^br^ cells. BMP-2 induces bone formation in heterogeneous osteosarcoma cells and BMP-2 may have a promising therapeutic role for treating human osteosarcoma by inducing differentiation along an osteogenic pathway.

## Introduction

Osteosarcoma (OS) is the most frequent primary bone malignancy comprising almost 60% of all bone sarcomas and a leading cause of cancer-related death among children, adolescents and young adults ( 1 ) . Despite modern multimodality therapies, long-term survival rates of ∼70% can be achieved only for those patients with resectable primary tumors and non-metastatic disease at initial diagnosis ( 2 ) . OS is believed to originate from undifferentiated mesenchymal cells and consists of osteoblastic, chondroblastic and fibroblastic cells or their combination. These histological features suggest that OS may arise from impaired differentiation of these immature cells into more mature types, thus it has been recently suggested that OS can be regarded as a differentiation disease. Restoring defective differentiation and/or correction of this impairment may be able to regulate tumorigenicity or reduce malignancy and increase the efficacy of chemotherapy. Therefore, differentiation induction holds great potential as a new modality of cancer therapy ( 3 , 4 ) .

Bone morphogenetic proteins (BMPs), with >30 different isoforms in a variety of organisms, belong to the transforming growth factor (TGF)-β superfamily known to regulate cell proliferation, differentiation, apoptosis, chemotaxis, angiogenesis and to participate in the development of most tissues and organ in vertebrates ( 5 ) . BMP-2, a member of this large family of proteins, has been originally identified for its ability to induce bone and cartilage formation when implanted at non-bony sites *in vivo* ( 6 ). Similar to TGF-β, BMP-2 exerts its effect via specific serine-threonine kinase receptors, type 1A (BMPR1A), type 1B (BMPR1B) and type 2 (BMPR2). BMP receptor type 2 is activated upon BMP-2 binding and subsequently induces oligomerization of the receptor complex, resulting in phosphorylation of the type 1 receptor and recruitment of downstream signaling Sma- and Mad-related proteins (Smad1, Smad5 and Smad8). The phosphorylated Smad1/5/8 can bind to a common mediator Smad4 in a heterodimeric complex that is translocated to the nucleus where it induces the expression of responsive genes such as Runx-2 that mediate the osteogenic activity of BMP-2 ( 7 , 8 ) . In addition to the canonical Smad pathway, non-Smad pathways mitogen-activated protein kinase (MAPK) pathways including p38, c-jun-N-terminal kinase (JNK) and extracellular signal-regulated kinase (ERK1/2) pathway, may also play important roles in cell proliferation and differentiation ( 9 , 10 ) .

Recently, several lines of evidence have revealed BMP-2 signaling in cancer cells. Expressions of BMP-2 and BMP receptors have been found to be altered in many tumor types ( 11 – 17 ) . Bioengineered recombinant human BMP-2 (rhBMP-2) has been demonstrated to increase tumor growth of lung carcinoma ( 18 ) , pancreatic carcinoma ( 13 ) and prostate cancers cells in the absence of androgen ( 19 ) . However, the effect of rhBMP-2 on cancer cells remains controversial. Some studies have shown rhBMP-2 exerts inhibitory effects on many tumor cells including breast cancer, myeloma, gastric cancer, colon cancer and prostate cancer ( 19 – 24 ) . We have recently reported that rhBMP-2 inhibits the tumorigenicity of cancer stem cells with high aldehyde dehydrogenase (ALDH) activity (ALDH ^br^ cells) derived from human OS xenografts ( 25 ) . We also reported that rhBMP-2 inhibits tumor growth and induces bone formation in human renal cell carcinoma cells ( 26 ) . These results led us to examine the possibility that BMP-2 induces bone formation in human OS cells.

In the present study, we sought to more extensively explore the effect of BMP-2 on heterogeneous population of ALDH ^br^ cells and their progenies with low ALDH activity (ALDH ^lo^ cells) derived from human OS xenografts. Our findings that BMP-2 differentially induces the expression of osteogenic marker genes in ALDH ^br^ and ALDH ^lo^ cells mediated by Smad signaling pathway provide a striking implication with the use of BMP-2 to restrict human OS expansion.

## Materials and methods

### Human OS cell culture

Human OS OS99-1 cell line originally derived from a highly aggressive primary human OS ( 27 ) was a generous gift from Dr Sheila M. Nielsen-Preiss (Montana State University, Bozeman, MT, USA). Cells were routinely cultured in Dulbecco’s modified Eagle’s medium (DMEM)/F12 medium (Gibco, Carlsbad, CA, USA) supplemented with 10% fetal bovine serum (Gibco) in a humidified atmosphere of 5% CO _2_ in air at 37°C and used when in the log phase of growth.

### Xenografts, tumor dissociation, ALDEfluor cell analysis and flow cytometry

Immunodeficient non-obese diabetic (NOD)/severe combined immunodeficient (SCID) (NOD/SCID) mice (5- to 6-week-old) were purchased from Harlan Laboratories (Harlan Laboratories, Indianapolis, IN, USA). All animal studies were performed according to protocol approved by the Institutional Animal Care and Use Committee of the University of Michigan. Xenografts and basic experimental procedures for tumor dissociation, ALDEfluor cell analysis and flow cytometry to isolate ALDH ^br^ cells and ALDH ^lo^ cells were detailed elsewhere ( 25 , 28 ) .

### Semi-quantitative real-time polymerase chain reaction (PCR)

To test the expression of BMP receptors in ALDH ^br^ cells and ALDH ^lo^ cells, total RNA was extracted and semi-quantitative PCR was run as described previously ( 25 , 28 ) .

### Quantitative real-time polymerase chain reaction (qPCR)

To further compare the expression of BMP receptors in freshly sorted ALDH ^br^ cells and ALDH ^lo^ cells, quantitative real-time PCR of BMPR1A (Hs01034913_g1), BMPR1B (Hs00176144_m1) and BMPR2 (Hs00176148_m1) and β-actin gene expression were run in triplicate using Eppendorf Mastercycler Realplex Detection System (Eppendorf, Germany). All primers were designed and purchased from Applied Biosystems (Life Technologies Corp., Carlsbad, CA, USA). To test the expression of osteogenic markers in sorted ALDH ^br^ and ALDH ^lo^ cells in response to BMP-2 (GenScript Corp., Piscataway, NJ, USA), freshly sorted cells were washed and cultured for expansion and then inoculated in a 6-well culture plate. After 24-h incubation, the medium was replaced with 1% serum-containing medium for 24 h and then replaced with 0 and 300 ng/ml BMP-2 diluted in 1% serum-containing medium. After 48 h total RNA was extracted as described above. Quantitative real-time PCR of osteogenic markers Runx-2 (Hs00231692_m1), Osx (Hs018666874_s1), ALP (Hs01029144_m1) and collagen type I (Hs00164004_m1) and β-actin gene expression were run in triplicate as described previously ( 25 , 28 ) .

### Western blot analysis

Freshly sorted cells were washed and cultured for expansion and then inoculated, at 5×10 ^5^ cells, in a 10-cm culture dish and grown to ∼80–90% confluence. The medium was replaced with 1% serum-containing medium for 24 h and then replaced with 0 and 300 ng/ml BMP-2 diluted in 1% serum-containing medium for the time indicated. Cells were lysed as previously described ( 26 ) . The protein concentrations were then measured using BCA protein assay kit (Thermo Scientific, Pittsburgh, PA, USA). Next, the protein lysates were separated by sodium dodecyl sulfate-polyacrylamide (SDS-PAGE) gel electrophoresis and then transferred onto hybond-C pure nitrocellulose membrane (Amersham, Piscataway, NJ, USA). Membranes were blocked with TBS containing 0.1% Tween-20 containing 5% non-fat dry milk and then incubated with primary antibody overnight. The primary antibodies were as follows: anti-phospho-Smad1/5/8, anti-phospho-ERK1/2, anti-anti-phospho-p38MAPK and anti-GAPDH (Cell Signaling Technology, Danvers, MA, USA). After washing with TBS with Tween-20, the secondary antibodies were added. Finally, the proteins were visualized with the ECL chemiluminescence system (Amersham).

### In vivo co-treatment experiments

Freshly sorted ALDH ^br^ cells and ALDH ^lo^ cells (1×10 ^4^ ) treated with BMP-2 or vehicle control were subcutaneously injected into right and left lower abdominal area of NOD/SCID mice. A more detailed description of this procedure can be found in Wang *et al* ( 26 ) . Tumor growth was monitored weekly for 12 weeks. Tumors formed were removed and a portion of each tumor was processed for histological analysis.

### Bone formation analysis

The mice were sacrificed and specimens harvested 12 weeks after implantation. Radiographs were obtained using Faxitron X-ray unit (Field Emission Corp., McMinniville, OR, USA). For microcomputed tomograpgy (micro-CT) analysis, specimens were scanned at 8.93 *μ* m voxel resolution on a micro-CT scanner (EVS Corp.), with a total of 667 slices per scan. GEMS MicroView software (GE Healthcare Biosciences) was used to make a three-dimensional reconstruction from the set of scans. Three samples per treatment were assessed. A cylindrical region of interest (ROI) was concentrically positioned over the defect site and kept constant for all the samples. The total volume of newly-formed bone within the ROI was measured by assigning a predetermined threshold and bone mineral content and was recorded as previously described ( 29 ) .

### Histological analysis

For histomorphometry, specimens were stained with haematoxylin and eosin (H&E) and Masson’s trichrome staining to show collagen type I protein in the newly formed bone. Undecalcified sections were stained with von-Kossa staining to identify the calcification during osteogenesis in the tumor.

### Statistical analysis

Data were expressed as mean ± SD. Statistically significant differences were determined by two-tailed Student’s t-test and defined as P<0.05.

## Results

### BMP receptor mRNA expression in freshly sorted ALDH ^br^ and ALDH ^lo^ cells

**Figure 1.**
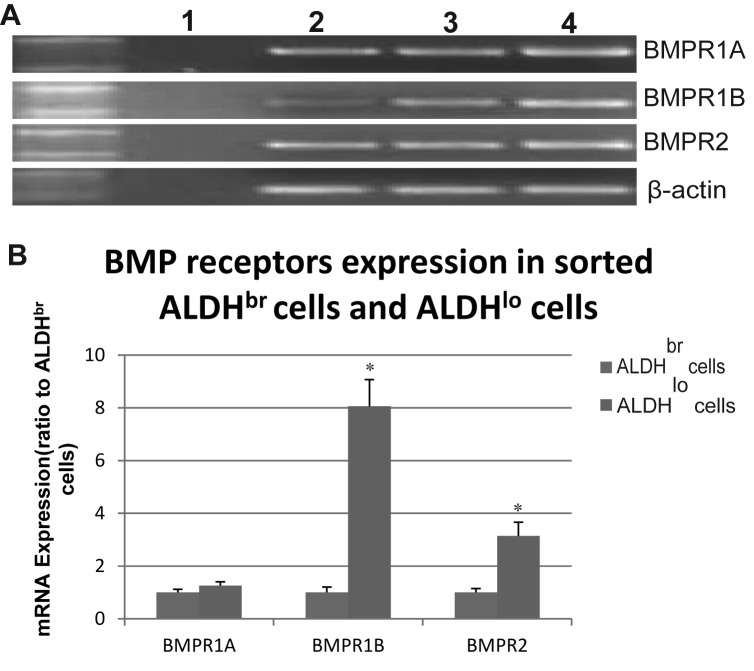
Expression of BMP receptors in ALDH ^br^ and ALDH ^lo^ cells. (A) Messenger RNA transcripts for BMP receptors. Lane 1, negative control; lane 2, ALDH ^br^ cells; lane 3, ALDH ^lo^ cells; lane 4, MCF7 cells (positive control). (B) Quantitative mRNA expression of BMP receptors in freshly sorted ALDH ^br^ and ALDH ^lo^ cells. Gene expression levels were normalized to β-actin. There was significantly higher mRNA expression of BMPR1B and BMPR2 in ALDH ^lo^ cells compared with that in ALDH ^br^ cells (P<0.05) and the BMPR1B expression in ALDH ^lo^ cells showed ∼8-fold higher than that in ALDH ^br^ cells. The mRNA expression of BMPR1A was higher in ALDH ^lo^ cells compared with that in ALDH ^br^ cells, but there was no significant difference between the two cell types (P>0.05).

We first examined the mRNA expression of the BMP type 1 and 2 receptors in freshly sorted ALDH ^br^ and ALDH ^lo^ cells derived from OS99-1 xenografts. As shown in Fig. 1 , all BMP-2 receptors were expressed in ALDH ^br^ and ALDH ^lo^ cells. Quantitative RT-PCR revealed that there was significantly higher mRNA expression of BMPR1B and BMPR2 in ALDH ^lo^ cells compared with that in ALDH ^br^ cells (P<0.05) ( Fig. 1B ) and the BMPR1B expression in ALDH ^lo^ cells was around 8-fold higher than that in ALDH ^br^ cells. The mRNA expression of BMPR1A was higher in ALDH ^lo^ cells compared with that in ALDH ^br^ cells, but there was no significant difference between the two cell types ( Fig. 1B ).

### Expression of osteogenic marker genes induced by BMP-2

**Figure 2.**
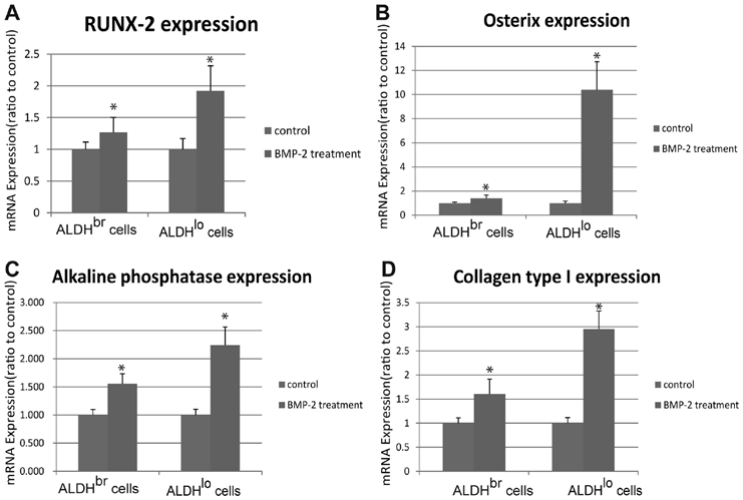
BMP-2 upregulates expression of osteogenic markers in ALDH ^br^ and ALDH ^lo^ cells. Quantitative mRNA expression of Runx-2, Osx, ALP and collagen type I genes in ALDH ^br^ and ALDH ^lo^ cells treated with 300 ng/ml of BMP-2 for 48 h. Gene expression levels were normalized to β-actin. Both ALDH ^br^ cells and ALDH ^lo^ cells treated with BMP-2 had significantly higher expression of Runx-2 (A), Osx (B), ALP (C) and collagen type I (D) than the cells treated with the same volume of vehicle (P<0.05). BMP-2 induced higher transcription of osteogenic markers in ALDH ^lo^ cells than in ALDH ^br^ cells and especially Osx expression in ALDH ^lo^ cells treated with BMP-2 showed ∼10-fold higher than that in ALDH ^lo^ cells treated with vehicle control. Each experiment was performed 3 times; representative examples are shown.

BMP-2 has been shown to act as a potent inducer of osteogenic differentiation ( 30 ) . Based on our previous report that BMP-2 significantly inhibits the growth of ALDH ^br^ cells at 300 ng/ml for 48 h ( 25 ) , we chose to treat ALDH ^br^ and ALDH ^lo^ cells with BMP-2 at the same dose for the following experiment. As shown in Fig. 2 , ALDH ^br^ and ALDH ^lo^ cells treated with BMP-2 had significantly higher expression of Runx-2, Osx, ALP and collagen type I than the cells treated with the same volume of vehicle (P<0.05). In addition, BMP-2 induced higher transcription of osteogenic markers in ALDH ^lo^ cells than in ALDH ^br^ cells and Osx expression in ALDH ^lo^ cells treated with BMP-2 showed around 10-fold higher than that in ALDH ^lo^ cells treated with vehicle control ( Fig. 2B ).

### BMP-2 activates Smad pathway in sorted ALDH ^br^ and ALDH ^lo^ cells

**Figure 3.**
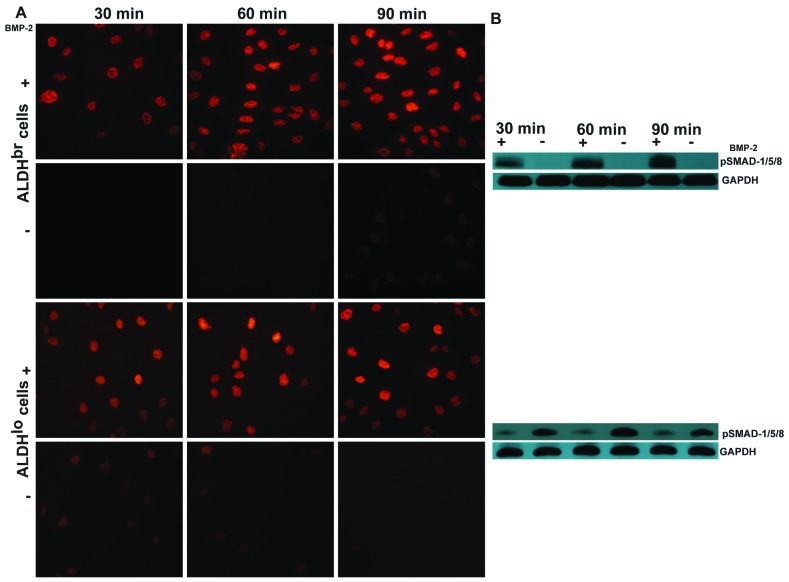
BMP-2 activates Smad1/5/8 in ALDH ^br^ and ALDH ^lo^ cells. Phosphorylated Smad1/5/8 in ALDH ^br^ and ALDH ^lo^ cells treated with 300 ng/ml BMP-2 or vehicle control was detected either by immunofluorescent staining or western blot analysis. (A) Immunofluorescence staining of phosphorylated Smad1/5/8 revealed activated Smad proteins were clearly located in the nuclei of ALDH ^br^ and ALDH ^lo^ cells treated with BMP-2 at 300 ng/ml for 30, 60 and 90 min, respectively. (B) Western blot analysis of phosphorylated Smad1/5/8 demonstrated that the highest levels of activated Smad proteins in ALDH ^br^ cells at 90 min, while in ALDH ^lo^ cells at 60 min. Equally loaded protein amounts were demonstrated by detection of GAPDH.

To determine whether BMP-2 signaling pathways were functional in ALDH ^br^ and ALDH ^lo^ cells, we first stimulated cells with BMP-2 and examined the phosphorylation and nuclear translocation of Smad1/5/8, since BMP-2 is thought to predominantly act through the activation of these transcription factors ( 31 ) . Both ALDH ^br^ cells and ALDH ^lo^ cells responded to BMP-2 treatment in cell culture. Immunofluorescence staining of phosphorylated Smad1/5/8 using an antibody which specifically recognizes the phosphorylated forms demonstrated activated Smad proteins were clearly located in the nuclei of ALDH ^br^ and ALDH ^lo^ cells treated with BMP-2 at 300 ng/ml for 30, 60 and 90 min, respectively ( Fig. 3A ). The ability of BMP-2 to phosphorylate Smad1/5/8 was then confirmed by western blot analysis after cells were treated with BMP-2 or vehicle control. As shown in Fig. 3B , western blot analysis of phosphorylation of Smad1/5/8 revealed the highest levels of activated Smad proteins in ALDH ^br^ cells at 90 min, while in ALDH ^lo^ cells at 60 min. These results revealed that BMP receptors are functional and BMP-2 can induce a classical Smad signaling pathway in ALDH ^br^ and ALDH ^lo^ cells.

### BMP-2 activates ERK and p38 MAPKS pathways in sorted ALDH ^lo^ cells

**Figure 4.**
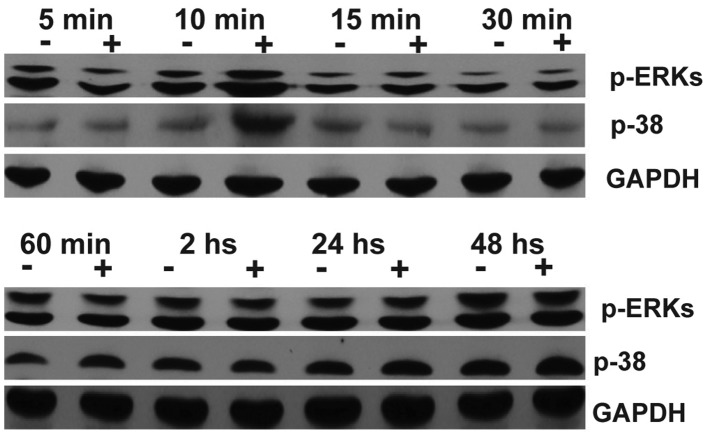
BMP-2 activates ERK1/2 and p38 MAPK pathways in ALDH ^lo^ cells. Constitutive phosphorylation of ERK and p38 MARKs was visible in ALDH ^lo^ cells and a slight transient increase was induced in the cytoplasm after 10 min of BMP-2 treatment. Equally loaded protein amounts were demonstrated by detection of GAPDH.

BMP-2 has also been shown to induce osteoblastic differentiation through extracellular signal-regulated kinase 1/2 (ERK1/2) and p38 mitogen-activated protein kinase (MAPK) pathways in human osteoblast cells ( 32 ) . To determine if BMP-2 stimulation leads to MAPK activation in sorted ALDH ^br^ and ALDH ^lo^ cells, we examined the phosphorylation of p38 and ERK1/2 using a specific antibody that recognizes phosphorylated serine sites. No BMP-2 induced phosphorylation of p38 and ERK1/2 was observed in sorted ALDH ^br^ cells (not shown). However, in ALDH ^lo^ cells, constitutive phosphorylation of ERK and p38 MARKs was visible and a slight transient increase was induced in the cytoplasm after 10 min of BMP-2 treatment ( Fig. 4 ).

### BMP-2 induces bone formation in sorted ALDH ^br^ cells and ALDH ^lo^ cells in vivo

**Figure 5.**
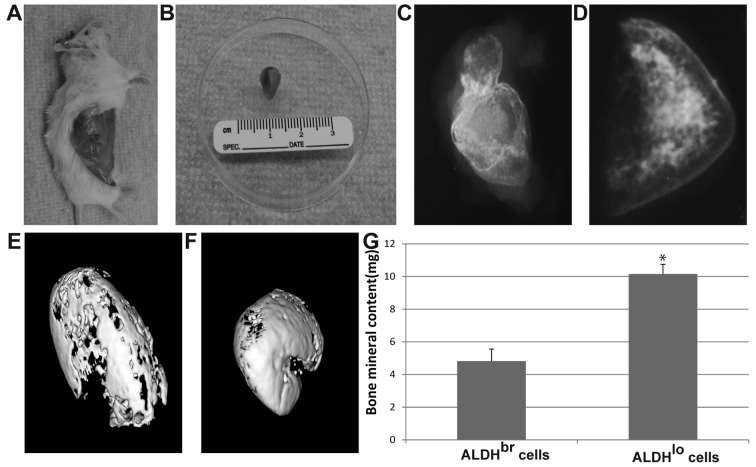
BMP-2 induces bone formation in ALDH ^br^ and ALDH ^lo^ cells *in vivo* . (A and B) Representative bone formation of sorted ALDH ^lo^ cells with BMP-2 treatment at the injection site in a NOD/SCID mouse. (C) Representative bone formations generated from ALDH ^br^ cells (C and E) and ALDH ^lo^ cells (D and F) with BMP-2 treatment were clearly demonstrated by radiograph and micro-CT measurements of bony ossicles. Images are representative of n=4 animals per group. (G) Bone formation induced by BMP-2 in ALDH ^lo^ cells showed significantly more bone mineral content compared to that in ALDH ^br^ cells (P<0.05).

**Figure 6.**
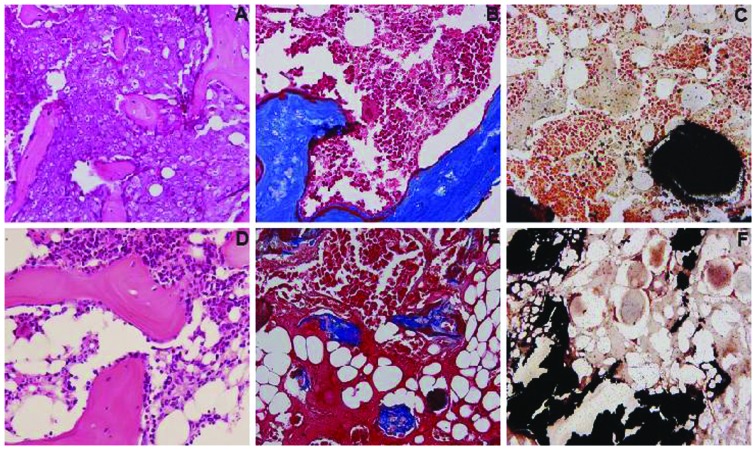
Bone formations in ALDH ^br^ and ALDH ^lo^ cells *in vivo* were confirmed by histological analysis. Representative bone formation generated from ALDH ^br^ cells treated with BMP-2 did not reveal bone marrow by H&E staining (A), blue collagen was presented by Masson’s trichrome staining (B) and calcified bone matrix was detected by von-Kossa staining (C). In ALDH ^lo^ cells, H&E staining revealed bone marrow which included blood vessels, fat and hematopoietic cells in the bone formation (D), Masson’s trichrome staining showing blue collagen (E) and Von Kossa staining showing calcified bone matrix (F).

We next injected freshly sorted ALDH ^br^ and ALDH ^lo^ cells with BMP-2 treatment or vehicle control subcutaneously into NOD/SCID mice. Both ALDH ^br^ cells and ALDH ^lo^ cells were induced to form bone in the mouse ectopic subcutaneous model. The bone formation induced by the addition of 30 *μ* g/animal of BMP-2 with ALDH ^lo^ cells was palpable within 1–2 weeks after injection ( Fig. 5A and B ), while the bone formation induced by ALDH ^br^ cells was palpable at 7–8 weeks. This was verified by 2D X-ray analyses ( Fig. 5C and D ) and 3D micro-CT ( Fig. 5E and F ). We chose a pre-determined threshold that resembles denser, cortical bone as this would be of direct clinical relevance. Bone formation induced by BMP-2 in ALDH ^lo^ cells showed significantly more bone mineral content compared to that in ALDH ^br^ cells (P<0.05) ( Fig. 5G ). Hematoxylin and eosin staining revealed that bone marrow which included blood vessels, fat and hematopoietic cells was observed in the bone formation induced in ALDH ^lo^ cells, which was also confirmed by Masson’s trichrome staining and von-Kossa staining ( Fig. 6D–F ). However, no marrow was found in the bone formation induced in ALDH ^br^ cells ( Fig. 6A–C ).

## Discussion

OS is the most common malignant tumor of the bone in the pediatric age group, with an incidence of 8.7 per million in children and adolescent under the age of 20 years ( 33 ) . OS is a clinically and molecularly heterogeneous group of malignancies characterized by varying degrees of mesenchymal differentiation. It has been proposed to be a differentiation-flawed disease and believed to arise from mesenchymal stem cells or osteoprogenitor cells resulting from a disruption in the osteoblast differentiation ( 34 ) . Although modern multi-modality therapies have improved the 5-year survival rate of OS patients, recurrent and/or metastatic OS tumors are more aggressive and usually resistant to conventional cancer therapies. Identification of the critical differentiation defects in OS tumors may lead to a rational design of therapeutic strategies that can induce terminal differentiation of OS cells through alternative differentiation pathways and/or bypassing the differentiation defects ( 34 ) .

Bone morphogenetic proteins (BMPs) were originally identified as osteoinductive cytokines to induce the entire cascade of cartilage and bone formation *in vivo* ( 35 ) . There are >30 isotypes BMPs, with BMP-2 and BMP-4 having 92% of homology. Studies on a variety of human cancer cells revealed that BMPs produce a complex set of effects in cancer, in which they can function as either protumorigenic oncogene or antitumorigenic tumor suppressor, depending on the stage of disease ( 36 ) . BMP-4 has been reported to induce differentiation of brain tumor stem cells *in vivo* ( 37 ) . More recently, we have demonstrated that treatment of human OS-derived tumor-initiating cells with BMP-2 inhibits cell proliferation and importantly, reduces the ability to form tumors in immunodeficient mice ( 25 ) . We also found that BMP-2 has an inhibitory effect on human renal cell carcinoma cells and induces bone formation ( 26 ) . BMP-2 has also been reported to induce *in vitro* differentiation of canine osteosarcoma cells ( 38 ) . However, little is known about bone formation of BMP-2 in human OS cells.

It has been reported that BMP signaling for the growth and differentiation of normal or neoplastic cells is dependent on its receptors ( 39 ) . There are currently 3 characterized BMP receptors: BMPR1A, BMPR1B and BMPRR2. Activation of the BMP receptor complex initiates intracellular signaling transduction ( 6 ) . In the present study, using regular RT-PCR, we determined that all 3 types of BMP receptors were expressed in freshly sorted ALDH ^br^ and ALDH ^lo^ cells derived from OS99-1 xenografts, suggesting that BMP-2 could bind to its receptors and activate cell signaling to affect osteosarcoma cell activities. By using quantitative RT-PCR, we further demonstrated that there was significantly higher mRNA expression of BMPR1B and BMPR2 in ALDH ^lo^ cells compared with that in ALDH ^br^ cells and the BMPR1B expression in ALDH ^lo^ cells showed ∼8-fold higher than that in ALDH ^br^ cells. These results indicate higher expression of BMPR1B in ALDH ^lo^ cells might correlate with its differentiated property as we have previously shown that ALDH ^br^ cells from human osteosarcoma OS99-1 xenografts has been identified as cancer stem cells and have the capability to produce differentiated progeny ALDH ^lo^ cells ( 28 ) . In agreement with this view, previous studies have shown that the expression of BMPR1B and BMPR2 in benign ovarian tumors and normal ovarian tissue was significantly higher than those in ovarian cancer tissue ( 40 ) . In addition, Hall and Miller ( 41 ) revealed that increased expression of BMPR1B in response to BMP2/4 promotes neuronal and astrocytic differentiation of neural stem cell. Forced expression of BMPR1B either by transgene expression or demethylation of the promoter restores differentiation capabilities and induces loss of their tumorigenicity in glioblastoma tumor initiating cells ( 42 ) . Thus, our findings further support that non-tumorigenic ALDH ^lo^ cells are more differentiated progeny cells compared with tumorigenic ALDH ^br^ cells. Higher expression of BMPR1B and BMPR2 in non-tumorigenic ALDH ^lo^ cells suggests that BMP-2 may act through its main receptors, BMPR1B and BMPR2, to induce osteogenic differentiation in non-tumorigenic ALDH ^lo^ cells.

BMP-2 has been shown to play important roles in the regulation of differentiation of many different cell types along osteoblastic pathways ( 43 , 44 ) . BMP-2 target genes include a growing number of tissue-determining transcription factors that promote differentiation of different cell types toward the osseous cell phenotypes ( 45 ) . The runt homology domain factor Runx-2 (Cbfa1) and Osx have been widely accepted as osteoblast-specific transcriptional factors along osteoblastic pathways since neither Runx-2 nor Osx null mice form mature osteoblasts ( 46 ) . Osx expression is more restricted to osteoblasts than Runx-2 ( 46 ) . ALP is an early osteoblast marker and collagen type I comprises 85–90% of the total bone matrix ( 47 ) . Runx-2 and collagen type I are known to be upregulated by BMP-2 in human prostate cancer cells, osteosarcoma cells renal carcinoma cells ( 21 , 25 , 26 ) . Therefore, using quantitative RT-PCR, we found that Runx-2, Osx, ALP and collagen type I were significantly upregulated in sorted ALDH ^br^ and ALDH ^lo^ cells treated with BMP-2 at a concentration of 300 ng/ml for 48 h when compared with untreated controls. BMP-2 induced higher transcription of osteogenic markers in ALDH ^lo^ cells than in ALDH ^br^ cells and especially Osx expression in ALDH ^lo^ cells treated with BMP-2 showed ∼10-fold higher than that in ALDH ^lo^ cells treated with vehicle control. These results indicated that non-tumorigenic ALDH ^lo^ cells are more likely to be induced to express higher level of osteogenic markers than tumorigenic ALDH ^br^ cells. Our findings suggested that BMP-2 might induce human osteosarcoma cells to express an osteoblastic phenotype and thus activate osteogenic differentiation to form bone.

BMP-2 is thought to predominantly exert their effect via binding to 2 types of receptors, leading to downstream transduction of the BMP signal through phosphorylation of specific intracellular proteins called Smads ( 48 ) . To further explore whether or not BMP-2 signaling pathways were functional in ALDH ^br^ and ALDH ^lo^ cells, the phosphorylation and nuclear translocation of Smad1/5/8 were detected by immunostaining and western blot analysis using an antibody which specifically recognizes the phosphorylated Smad1/5/8. Our results demonstrated that the highest level of phosphorylation of Smad1/5/8 in ALDH ^br^ cells at 90 min and in ALDH ^lo^ cells at 60 min. The elevation of Smad activation at the 60- or 90-min time-point, before osteogenic markers Runx-2, Osx, ALP and collagen type I were significantly upregulated after 48 h of treatment, suggests that in transcription regulation of bone-related osteogenic markers gene expression by BMP-2 might be mediated via the Smads transducer. In addition, BMP-2 was shown to activate ERK1/2 and p38 MAPK pathways in ALDH ^lo^ cells, but not in ALDH ^br^ cells. This difference in the activation of MAPK pathways between ALDH ^lo^ cells and ALDH ^br^ cells may be due to the type of receptors expressed by the cells. Therefore, activation of MAPK pathways in ALDH ^lo^ cells by BMP-2 might have a synergetic effect in the induction of higher Osx mRNA expression since BMP-2 has been demonstrated to induce Osx expression through phosphorylation of p38 ( 45 ) . Further experiments are therefore required to understand the different gene expression between ALDH ^lo^ and ALDH ^br^ cells induced by BMP-2.

Determining the bone formation of BMP-2 in osteosarcoma cells *in vivo* is essential for determining the potential use of BMP-2 clinically because *in vitro* analysis does not always reflect exactly the *in vivo* situation. In the present study, BMP-2 was shown to induce bone formation in both tumorigenic ALDH ^br^ cells and non-tumorigenic ALDH ^lo^ cells. All animals receiving non-tumorigenic ALDH ^lo^ (1×10 ^4^ ) cells treated with 30 *μ* g BMP-2 per animal resulted in significant bone formation within 1–2 weeks after injection in NOD/SCID mice. The bone formation was further confirmed by radiograph and micro-CT and histopathological analysis of sections from bony tissues formed by ALDH ^br^ and ALDH ^lo^ cells treated with BMP-2. These findings are in agreement with previous results indicating that viral vector-induced expression of BMP-2 in a breast cancer cell line and a colon cancer cell line induces calcification of tumors to arrest tumor growth ( 49 ) . By contrast, Luo *et al* ( 30 ) reported that osteogenic BMPs promote tumor growth of human osteosarcoma. They infected osteosarcoma cells with adenoviral vectors to secret BMP-2 and BMP-9. Factors such as the concentration and distribution of these endogenous BMP compared to exogenous application of BMP-2 as done in this study may be the possible explanation for the proliferation of osteosarcoma cells observed in that investigation.

Our study is the first to provide evidence indicating that exogenous BMP-2 induces bone formation in human osteosarcoma cells. However, the use of only one cell line provides limited evidence. Further research using more cell lines and primary tumors is therefore necessary to confirm the findings of this study.

In conclusion, our findings suggest that BMP-2 can upregulate osteogenic markers Runx-2, Osx, ALP and collagen type I gene expression in both ALDH ^br^ cells and ALDH ^lo^ cells and this osteoinductive effect may be mediated by Smad signaling pathway. Subsequently, we demonstrated that BMP-2 induced bone formation *in vivo* . Our findings present evidence on a potential therapeutic application of exogenous BMP-2 on human osteosarcoma by inducing differentiation of tumorigenic cells along an osteogenic pathway.
